# *Origanum vulgare* Essential Oil Modulates the AFB1-Induced Oxidative Damages, Nephropathy, and Altered Inflammatory Responses in Growing Rabbits

**DOI:** 10.3390/toxins15010069

**Published:** 2023-01-12

**Authors:** Mona A. Hassan, Azza M. A. Abo-Elmaaty, Asmaa W. Zaglool, Sally A. M. Mohamed, Shimaa M. Abou-Zeid, Mayada R. Farag, Mahmoud Alagawany, Alessandro Di Cerbo, Mahmoud M. Azzam, Rashed Alhotan, Enas EL-Hady

**Affiliations:** 1 Department of Forensic Medicine and Toxicology, Faculty of Veterinary Medicine, Zagazig University, Zagazig 44519, Egypt; 2 Department of Pharmacology, Faculty of Veterinary Medicine, Zagazig University, Zagazig 44519, Egypt; 3 Animal Wealth Development Department, Faculty of Veterinary Medicine, Zagazig University, Zagazig 44519, Egypt; 4 Department of Histology and Cytology, Faculty of Veterinary Medicine, Zagazig University, Zagazig 44519, Egypt; 5 Department of Forensic Medicine and Toxicology, Faculty of Veterinary Medicine, University of Sadat City, Sadat City 6012201, Egypt; 6 Poultry Department, Faculty of Agriculture, Zagazig University, Zagazig 44519, Egypt; 7 School of Biosciences and Veterinary Medicine, University of Camerino, 62024 Matelica, Italy; 8 Department of Animal Production, College of Food & Agriculture Sciences, King Saud University, Riyadh 11451, Saudi Arabia; 9 Department of Anatomy and Embryology, Faculty of Veterinary Medicine, Zagazig University, Zagazig 44519, Egypt

**Keywords:** Marjoram essential oil, AFB1, rabbits, kidney, oxidative damage

## Abstract

The current study was performed to investigate the toxic effects of aflatoxin B1 (AFB1) through the evaluation of kidney function tests and histopathological examination of renal tissues, targeting the therapeutic role of Marjoram (*Origanum vulgare* essential oil-OEO) in improving health status. Forty-eight New Zealand Whites growing rabbits (four weeks old) weighing on average 660.5 ± 2.33 g were randomly and equally distributed into four groups, each of which had four replicas of three animals as the following: Control group (only basal diet), AFB1 group (0.3 mg AFB1/kg diet), OEO group (1 g OEO/kg diet) and co-exposed group (1 g OEO/kg + 0.3 mg AF/kg diet). Our study lasted eight weeks and was completed at 12 weeks of age. The results revealed that OEO decreased the toxic effects of AFB1 in rabbit kidneys by substantially reducing the cystatin C levels in the AFB1 group. Additionally, OEO decreased oxidative stress and lipid peroxidation levels in the co-exposed group. Moreover, OEO reduced DNA damage and inflammatory response in addition to the down-regulation of stress and inflammatory cytokines-encoding genes. Besides, OEO preserved the cytoarchitecture of rabbits’ kidneys treated with AFB1. In conclusion, *O. vulgare* essential oil supplementation ameliorated the deleterious effects of AFB1 on the rabbits’ kidneys by raising antioxidant levels, decreasing inflammation, and reversing oxidative DNA damage.

## 1. Introduction

Mycotoxins are fungi’s corrosive and cytotoxic metabolites and are considered widespread pollutants in the feed materials [1,2]. According to statistics, one-fourth of the feed components produced globally contain mycotoxins [3]. Mycotoxin contamination of forage and feed components is a serious issue that requires more attention. Mycotoxins are produced mainly by *Aspergillus flavus*, *Aspergillus parasiticus*, and *Aspergillus fumigatus* and Aflatoxins are thought to be the most harmful ones [4,5,6].

In developing counties like Egypt, there is a significant potential for the production of rabbits. The rabbit requires little area and little capital investment, and it grows quickly and has a high capacity for reproduction. Since rabbits have a functional caecum, they can naturally use the plentiful fibrous agricultural byproducts and herbaceous plants as food [7]. The presence of aflatoxins in feeds is a hazard to rabbit performance.

Despite the infrequent outbreaks of acute aflatoxins toxicity, animals are the most susceptible population, exposed to high accumulations of aflatoxins through the intake of contaminated feedstuffs [8].

Due to its broad toxicity, aflatoxin B1 (AFB1) has attained great attention. AFB1 was graded as a Group A carcinogen by the WHO in 1993, and its consumption in rabbits is related to a high hazard for liver and kidney cancer [9]. Additionally, AFB1 can potentially cause growth impairment, malnutrition, and immunosuppression, particularly in young animals [10].

Essential oils (EOs) are aromatic compounds derived from plant sources and are known for their high volatility and complicated structure. They are heavily used in pharmaceutical, agricultural, nutritional, and cosmetic industries because of their well-known antibacterial, antifungal, and antioxidant properties [11,12,13]. Concerning antifungal properties, various types of research have shown that essential oils can prevent the spread of a variety of fungi in foodstuffs, including *Aspergillus* [14]. Due to its antifungal characteristics, oregano essential oil is one of the most researched essential oils [15,16].

Due to its flavor-adding capacity, *Origanum vulgare* L. (*Lamiaceae*) is one of the most popular condiments used in food and pet food preparation [16,17,18,19]. Additionally, it is widely used in the production of essential oils due to its antimicrobial characteristics, which include the presence of carvacrol and thymol, which are effective against different types of fungi [20]. Moreover, Origanum can be used as a detoxifying substance against aflatoxins, and this point should be investigated [21]. The current study’s objective is to ascertain if *O. vulgare* essential oil (OEO) may have any protective benefits against aflatoxins by investigating the harmful effects of aflatoxins on renal function, oxidative stress, inflammatory responses, and the histological structure of rabbits’ kidneys.

## 2. Results

### 2.1. Effects on Kidney Function Markers

Urea and creatinine levels in the serum of the AFB1-exposed group were considerably higher than those in the control group, as indicated in Table 1. Urea and creatinine levels did not significantly differ from the control after using OEO supplements. Additionally, the urea levels were still higher than the control values in the AFB1 + OEO group, while the creatinine levels were stable.

The total and direct bilirubin concentrations in the AFB1-supplemented group were significantly greater than in control. In the OEO group, total and direct bilirubin levels did not alter significantly. In the AFB1 + OEO group, total bilirubin was higher than the control, but direct bilirubin remained steady.

Regarding glomerular filtration rate (GFR), Beta-2 microglobulin levels were not significantly altered in any treatment group. The findings of cystatin C indicated that aflatoxins increase their level compared to the control. At the same time, it remains constant in the OEO and AFB1 + OEO groups compared to the control (Table 1).

### 2.2. Effects on Oxidative Stress Biomarkers in Kidney

Table 2 lists the values of several antioxidant enzymes and oxidative stress indicators in the kidney of rabbits. Superoxide-dismutase (SOD) activity levels did not significantly alter in any treatment group relative to the control group.

Catalase activity was considerably higher in the OEO group but lower in the AFB1 and AFB1 + OEO-exposed groups compared to the control. GSH levels in the AFB1 and AFB1 + OEO groups significantly decreased, remaining constant in the OEO groups compared to the control.

Concerning oxidative stress biomarkers, lipid peroxidation, as demonstrated by the formation of MDA, was significantly elevated in the AFB1 group relative to the control. At the same time, there were no significant changes in MDA levels among the OEO and AFB1 + OEO groups.

Compared to the control, the AF-treated group showed a substantially (*** *p* < 0.001) higher level of protein carbonyl (PC) production, a marker of oxidative protein damage. However, there were no significant differences between the control, OEO, or AFB1 + OEO groups.

Regarding DNA oxidation biomarkers, our findings showed no observable differences in the serum level of 8-OHdG between control, OEO, or AFB1 + OEO groups. The 8-OHdG content was significantly higher in the AFB1 group than in the control and experimental groups.

### 2.3. Effects on Inflammatory Biomarkers

Table 3 illustrates the effect of AFB1 and OEO supplementation on inflammatory biomarkers. TNF-α levels increased dramatically in response to AFB1 administration alone compared to the control group. Co-supplementation of OEO with AF significantly decreased TNF-α levels to the control value. Nitric oxide (NO) levels did not significantly alter in none of the treatment groups compared to the control. The levels of TGF- β1 and VEGF were significantly (*** *p* < 0.001) increased in the AFB1 group. In contrast, co-treatment of OEO with AFB1 significantly decreased the TGF-β1 and VEGF levels more than AFB1 alone; however, it did not reach the control values.

### 2.4. Effects on Stress and Inflammatory Cytokines-Encoding Genes

**Figure 1:**
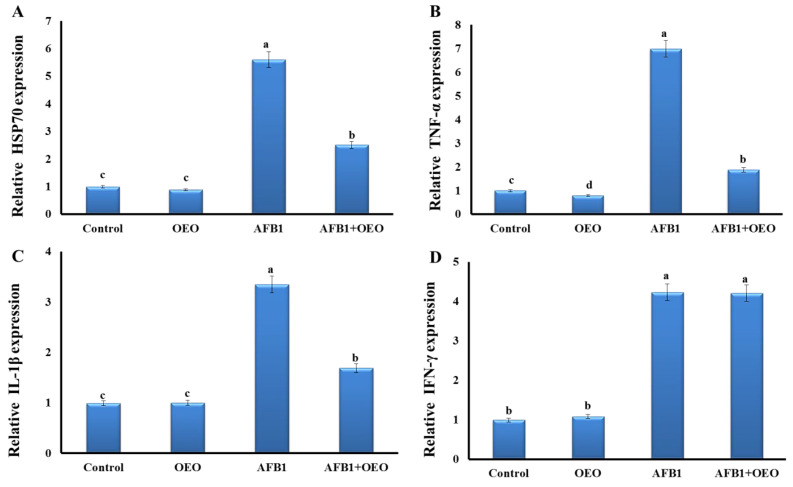
Effect of supplementation of OEO on the expression pattern of stress-related genes (**A**) and pro-inflammatory cytokines (**B**): TNFα; (**C**): IL-1β, and (**D**): IFN-γ) in the kidney of rabbits exposed to AFB1. Values are mean ± SE; bars that are not sharing a common superscript letter (a,b,c,d) differ significantly at *p* < 0.05.

As depicted in Figure 1, our findings demonstrated that most investigated genes had their expression levels significantly rise due to exposure to AFB1-contaminated feed.

Inflammatory markers expression significantly increased as a result of AFB1 exposure. There is an upregulation in the TNF-α gene expression in the AFB1 group, while there is downregulation in the OEO group. In the AFB1 + OEO group, a significant increase in TNF-α gene expression was observed.

Expression levels of IL-1β and HSP70 genes were significantly increased in the AFB1 group compared to the control. OEO alone did not affect these markers but caused a decrease in the expression of IL-1β and HSP70 genes compared to the AFB1 group.

Regarding IFN-γ gene expression, there is a significant increase in the AFB1 and AFB1 + OEO groups, while there is no change in the OEO group compared to the control.

### 2.5. Bioaccumulation of AFB1 Residues in Kidney Tissue

**Figure 2:**
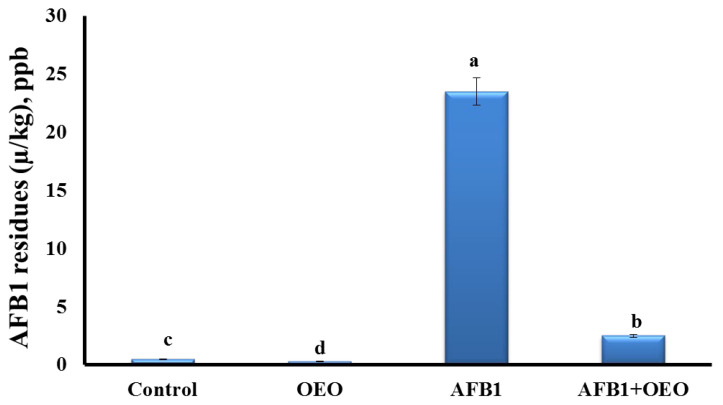
Effect of supplementation of OEO on the bioaccumulation of AFB1 residues in the kidney of rabbits. Values are mean ± SE; bars that are not sharing a common superscript letter (a,b,c,d) differ significantly at *p* < 0.05.

Analysis of AFB1 residues in the kidney tissue of control and treated rabbits are represented in Figure 2.

The highest concentration of aflatoxin residues was detected in the kidney of AFB1-exposed rabbits. OEO caused a significant decrease in the residual accumulation of AFB1 in the co-exposed group. However, the OEO group showed the lowest level of AFB1 residues among all the groups.

### 2.6. Histopathological Findings

**Figure 3:**
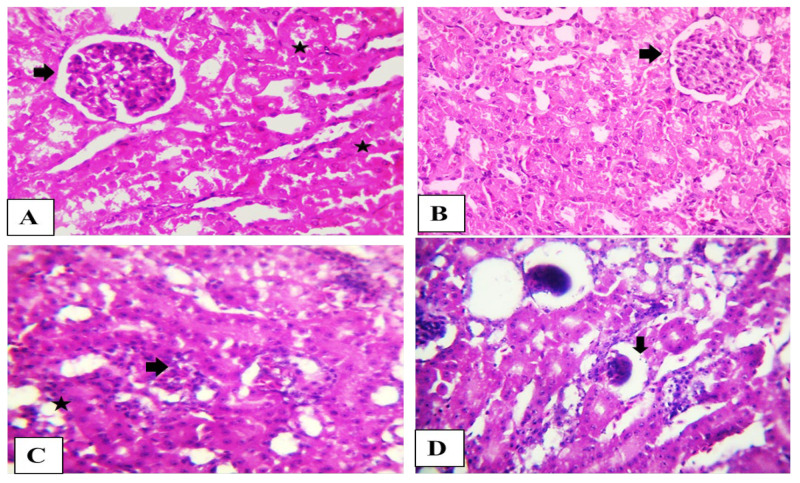
(**A**) Photomicrograph of the rabbit’s kidney (Control) and (**B**) (OEO) showing normal glomerular tufts and cellularity (arrow) besides normal proximal renal tubules (stars). (**C**) Photomicrograph of the rabbit’s kidney (Aflatoxin) showing disorder of glomeruli (arrows) characterized by the disappearance of Bowman’s spaces beside degenerated renal tubule epithelium (star). (**D**) Photomicrograph of the rabbit’s kidney (Aflatoxin + OEO) showing shrunken glomeruli (arrow). Scale bar is 100 µm. H&E stain.

The kidney from the control and OEO rabbits showed normal histological structure. The rabbit’s kidney in the AFB1 group showed the disappearance of Bowman’s spaces in glomeruli, degeneration of renal tubules epithelium renal blood vessels fibrosis with vacuolated media. The kidneys in the combination group showed shrunken glomeruli (Figure 3).

## 3. Discussion

As a result of the ongoing growth in the incidence of aflatoxicosis in developing nations, it is currently a global public health issue [5,22].

The laboratory findings usually examine urea and creatinine levels to determine the integrity and functionality of the nephrons. We observed that the AFB1 group had higher urea and creatinine levels than the control group, demonstrating the adverse effects of aflatoxin on the kidneys. These observations concur with prior studies [23]. However, co-supplementation with OEO restored kidney functional biomarker activities and levels, indicating that OEO acted as a safeguard against aflatoxicosis in the rabbits’ kidneys. The abovementioned results are consistent with those of Zowail et al. [24].

We assessed the glomerular filtration rate, a valuable marker for evaluating renal functioning. It is crucial to determine the levels of several freely filtered low molecular weight proteins, which are more pertinent than assessing the concentration of creatinine alone, to determine the impairments in GFR. These proteins include beta-2 microglobulin and cystatin C [25]. In our study, The AF-treated rabbits had considerably higher blood levels of urea, creatinine, and cystatin C than the control. This suggested the presence of kidney and glomerular filtration dysfunction. This result was in accordance with Orsi et al. [26] and Abdou et al. [27].

Several studies have demonstrated that oxidative stress plays a critical pathogenic role in the tissue damage caused by AFB1 that results in inflammatory damage [28]. AFB1 generated free radicals that can cause oxidative damage to cells after being bio-transformed into the electrophilic, intermediate metabolite AFB1 8,9-epoxide [29]. The results of the current investigation show that exposure to AFB1 causes oxidative stress to be induced in the kidney of rabbits. This was demonstrated by the dramatically decreased activities of the antioxidant molecules CAT and GSH and the significantly elevated levels of MDA and PC activities. In prior findings, it was revealed that the administration of AFB1 to rats and mouse models lowered the activity of antioxidant enzymes [30]. Catalase and GSH levels were markedly elevated when OEO was concurrently administered to rabbits. Additionally, MDA and PC levels in the serum have significantly decreased. These findings imply that OEO is increasing the levels and activity of antioxidant molecules. Additionally, recent research has demonstrated that OEO is beneficial in delaying lipid oxidation [31,32] and acts as a good antioxidant [33].

Following exposure to mycotoxins, oxidative stress and inflammation are closely related [34]. AFB1 exposure has been associated with the release of pro-inflammatory cytokines (including TNF-α, IL-1, and IL-6) and inflammation-related tissue damage [35]. In our study, there is upregulation in the expression of TNF-α in the kidney tissue of rabbits in addition to increased expression levels of IL-1β, INF, and HSP70 genes. The first and most significant inflammatory mediator is TNF-α, which controls the metabolic reactions of another tissue and encourages the production of different cytokines during the inflammation process [36].

The alterations in the inflammatory cytokine expression levels in the rabbits’ kidneys showed that the AFB1 exposure caused inflammatory damage. OEO supplementation dramatically reduced TNF-α, TGF, and TGF-β1, suggesting that OEO has anti-inflammatory potential. For instance, Arranz et al. showed that OEO decreased the production of IL-1, TNF-α, and IL-6 in LPS-activated THP-1 macrophage cells of humans [37]. Furthermore, Han and Parker demonstrated that, on activated-primary human newborn fibroblasts, OEO dramatically reduced the levels of different inflammatory biomarkers such as MCP-1, ICAM-1, and VCAM-1 [38]. These observations indicated that the OEO might have anti-inflammatory potential.

The activation of programmed cell death by oxidative DNA damage is another crucial mechanism of AFB1-induced kidney injury [39,40,41]. DNA damage inside a tissue or an organ is linked to the production of 8-OHdG, which may cause the activation of p53 and apoptosis.

In the current study, DNA oxidation biomarker 8-OHdG was significantly elevated in the AFB1 group compared to the control, and co-supplementation with OEO has an anti-genotoxic effect. Previous studies in mice exposed to cyclophosphamide [42] and people exposed to radioiodine [43] found that using *Origanum vulgare* extract in the diet reduces genotoxicity.

The results of the histological analysis of the kidney tissue in the AFB1 group had deteriorated renal tubule epithelium as well as glomeruli abnormalities characterized by the loss of Bowman’s spaces, demonstrating fibrosis of the major renal blood arteries, provided additional validation for the development of inflammation and apoptosis by AFB1.

## 4. Conclusions

The present study’s findings established that AFB1 negatively affected kidney tissue structure and function and induced its accumulation in renal tissues, while the use of natural feed additives such as OEO ameliorated physiological functions and health aspects by reducing oxidative stress, increasing the levels of antioxidants, resolving inflammation, and reversing DNA damage.

Moreover, OEO could reduce the AFB1 residues suggesting that besides its powerful antioxidant activity, it could also act as a chelator and this dual beneficial action makes OEO a powerful candidate for protecting rabbits from environmental contaminants.

## 5. Materials and Methods

### 5.1. Chemicals

*O. vulgare* essential oil (OEO) was obtained from Elhawag Company for Natural Oils, Nasr City, and Cairo, Egypt (LOT#: 150/2/159). The bioactive components of OEO were identified by gas chromatography-mass spectroscopy (GC–MS) analysis [44] and are represented in Table 4.

AFB1 preparation and selection of the used concentration were based on the previous observations [6]. To obtain AFB1, *Aspergillus flavus* MD 341 was retrieved from the Central Lab. of Residues of Agricultural Products, Dokki, Egypt and incubated for 8 days in liquid media with 2% of yeast extract and 20% of sucrose. A reversed-phase column was utilized for the extraction, filtration, and quantitative HPLC analysis of aflatoxins. 45% methanol served as the mobile phase and was inoculated into the apparatus at a flow rate of 1 mL per minute. The analyses were performed using a fluorescence detector, and the column temperature was set to 40 °C. Aflatoxin standard was bought from Sigma-Aldrich (St. Louis, MO, USA). The media was found to contain only AFB1.

### 5.2. Animals and Experimental Design

Forty-eight male New Zealand Whites growing rabbits (4 weeks old) weighing on average 660.5 ± 2.33 g were obtained from the laboratory animal farm, Faculty of Veterinary Medicine, University, Zagazig, Egypt. Rabbits were randomly and equally distributed into four groups, each of which had four replicas of 3 animals. The duration of this study was eight weeks, so it was completed at 12 weeks of age. The investigated groups were as the following: control group (only basal diet), AFB1 group (0.3 mg AFB1/kg diet), OEO group (1 g OEO/kg diet), and Combination group (1 g OEO/kg + 0.3 mg AF/kg diet). Rabbits were housed in proper cages (50 cm × 40 cm × 30 cm). Every rabbit was raised under the same management and sanitary settings, and the nutritional requirements were fulfilled by the feed they were given (Table 5).

The animal study was reviewed and approved by the institutional Ethics Committee of Zagazig University, Zagazig, Egypt (ZU-IACUC/2/F/387/2022).

### 5.3. Sampling and Analysis

After the last treatment, rabbits (6 per group) were euthanized, and blood samples were taken in sterile tubes, allowed to be clotted, and after that, centrifuged at 4000 rpm for 10 min. The collected serum was kept at −20 °C until they were tested. We collect serum to detect kidney function markers. Other blood samples were collected on heparinized tubes for plasma collection for measuring inflammatory markers. Specimens from the kidney tissue were excised, washed with normal saline (0.9% NaCl), and preserved in formalin for histological analysis. For further biochemical analysis, another kidney sample set was frozen at −20 °C. After being instantly frozen in liquid nitrogen, the third group of kidney specimens was kept at −80 °C until the RNA extraction.

### 5.4. Antioxidant Biomarkers

Kidney tissues were homogenized (10% *w*/*v*) in potassium phosphate buffer solution (pH 7.4) for antioxidant analyses, followed by 15 min centrifugation at 3000 rpm. Following the manufacturer’s guidelines, the obtained supernatant was used to test the activity of superoxide dismutase (SOD), catalase (CAT), and the level of reduced glutathione (GSH) using commercial bio diagnostic kits from BioMérieux, Marcy-l’Etoile, France.

### 5.5. Oxidative Stress Biomarkers

Malondialdehyde (MDA), a biomarker of lipid peroxidation, and Protein Carbonyls (PC), a marker of oxidative protein damage, were assessed in renal tissues, while 8-hydroxy-2-deoxyguanosine (8-OHdG), a biomarker of DNA oxidative damage was measured in serum, using ELISA kits from MyBiosource.com, San Diego, CA, USA (Cat No., MBS040484, MBS1601647, and MBS726394, respectively) following the manufacturer’s instructions.

### 5.6. Glomerular Filtration and Kidney Function Markers

Beta-2 microglobulin (B2M) and cystatin C were determined by ELISA kits (Rabbit ELISA kits; MBS2602011 and MBS285072, respectively) from MyBioSource, San Diego, California. While kits for measuring urea, creatinine, and total and direct bilirubin were purchased from (Bio Med Diagnostic, Giza, Egypt).

### 5.7. Inflammatory Biomarkers

Using ELISA kits from MyBioSource, San Diego, California, Nitric oxide (NO), transforming growth factor-1 (TGF-1), and vascular endothelial growth factor (VEGF) were measured in plasma while tumor necrosis factor (TNF-α) was assessed in serum (Cat No. MBS8243214, MBS704933, MBS751261, and MBS2500169, respectively).

### 5.8. Vicam AflaTest Fluorometer Technique

Using the mycotoxin calibration standards, AflaTest was calibrated. The purified water (2 mL) and the plain reagent (1 mL methanol and 1 mL developer) should both read 0 ppb on the calibrated fluorometer to assure accuracy. Each sample (25 g) was blended at high speed for 1 min with 125 mL of methanol: water (60:40) and 5 g of sodium chloride. The fluted filter paper was used to filter the mixture, and the extract (20 mL) was diluted with purified water (20 mL). A 1.5 mL glass microfiber filter was used to filter the diluted extract into a clean container. Filtered extract (10 mL, represents 1 g sample) at a flow rate of nearly two drops/s was passed through the affinity column, followed by water (10 mL, 2 drops/s). The AFB1 was eluted with HPLC methanol (1 mL, 1 drop/s) and then collected in a glass cuvette (VICAM part # 34000). The eluate and developer (1 mL) were well combined before being added to the calibrated fluorometer.

### 5.9. Gene’s Transcriptional Analysis (qRT-PCR Study)

Total RNA was extracted from frozen kidney samples using the TRIzol reagent (easyREDTM, iNtRON Biotechnology, Gyeonggi-do, South Korea). The first-strand cDNA was produced from the isolated RNA using the Quantitect^®^ Reverse Transcription kit from Qiagen, Hilden, Germany. Following the instructions provided by the kit’s manufacturer, RNA extraction and cDNA synthesis were carried out. The forward and reverse sequences of the primers for the investigated genes, including the housekeeping gene β-actin, the heat shock protein-70 (HSP-70), interleukin-1β (IL-1β), interferon-(IFN-γ), and tumor necrosis factor (TNF-α), are listed in Table 6.

The qPCR analysis was carried out using the Rotor-Gene Q instrument and a QuantiTect^®^ SYBR^®^ Green PCR kit (Qiagen, Hilden, Germany) under the following thermocycler conditions: 10 min at 95 °C, then 40 cycles at 95 °C for 15 s, 60 °C for 30 s, and 72 °C for 30 s. To confirm the specificity of the PCR, melt-curve analysis was carried out. The comparative 2^−ΔΔCt^ methodology recommended by Livak and Schmittgen was used to determine the relative mRNA expression pattern for each gene [45].

### 5.10. Histopathological Examination

Specimens of the kidney were obtained and fixed in buffered formalin 20% for two days. The samples were embedded in paraffin following gradual dehydration in 70% to 100% ethanol. Subsequently cut into sections (5 μm), stained by hematoxylin and eosin (H&E), and the histopathological analysis was conducted with the aid of the Olympus BX51 light microscope, Tokyo, Japan [46].

### 5.11. Statistical Analysis

The general linear models’ approach, modified by SPSS for the user’s handbook using one-way ANOVA, was used to evaluate the data statistically. The post-hoc Newman-Keuls test (* *p* < 0.05) was used to assess whether or not there were differences between the treatments.

## Figures and Tables

**Table 1:** Effect of supplementation of OEO (1 g/kg diet) on kidney function markers of rabbits exposed to AFB1 (0.3 mg/kg diet) for eight weeks.

Items	Kidney Function Markers					
	Urea (mg/dL)	Creatinine (mg/dL)	Total Bilirubin (mg/dL)	Direct Bilirubin (mg/dL)	B2 Microglobulin (ng/mL)	Cystatin C (ng/mL)
Control	31.47 ± 0.93 ^c^	1.20 ± 0.01 ^b^	1.01 ± 0.00 ^c^	0.61 ± 0.01 ^b^	0.14 ± 0.00 ^a^	0.11 ± 0.00 ^b^
OEO	31.18 ± 0.63 ^c^	1.20 ± 0.01 ^b^	1.01 ± 0.01 ^c^	0.54 ± 0.03 ^b^	0.14 ± 0.00 ^a^	0.10 ± 0.00 ^b^
AFB1	64.26 ± 1.63 ^a^	1.84 ± 0.02 ^a^	1.82 ± 0.03 ^a^	0.87 ± 0.1 ^a^	0.14 ± 0.00 ^a^	0.39 ± 0.01 ^a^
AFB1 + OEO	43.59 ± 1.85 ^b^	1.20 ± 0.01 ^b^	1.34 ± 0.06 ^b^	0.64 ± 0.09 ^b^	0.14 ± 0.00 ^a^	0.12 ± 0.00 ^b^
*p*-value	<0.001	<0.001	<0.001	<0.001	0.859	<0.001

^a–c^ Different superscripts within each column are significantly different (*p* < 0.05).

**Table 2:** Effect of supplementation of OEO (1 g/kg diet) on antioxidants and oxidative stress biomarkers in the kidney of rabbits exposed to AFB1 (0.3 mg/kg diet) for eight weeks.

Items	Antioxidant and Oxidative Stress Parameters					
	SOD (μg/g tissue)	CAT (μg/g tissue)	GSH (μg/g tissue)	MDA (nmol/g tissue)	PC (nmol/g tissue)	8-OHdG (ng/mL)
Control	0.20 ± 0.01	0.54 ± 1.90 ^b^	0.21 ± 0.00 ^a^	0.17 ± 0.00 ^b^	5.58 ± 0.08 ^b^	0.23 ± 0.01 ^b^
OEO	0.20 ± 0.01	0.80 ± 1.01 ^a^	0.20 ± 0.00 ^a^	0.11 ± 0.00 ^c^	5.35 ± 0.07 ^b^	0.19 ± 0.01 ^b^
AFB1	0.20 ± 0.03	0.30 ± 0.01 ^c^	0.15 ± 0.00 ^b^	0.46 ± 0.00 ^a^	8.92 ± 0.15 ^a^	0.35 ± 0.00 ^a^
AFB1 + OEO	0.20 ± 0.01	0.28 ± 0.01 ^c^	0.07 ± 0.01 ^c^	0.17 ± 0.01 ^b^	5.57 ± 0.04 ^b^	0.24 ± 0.01 ^b^
*p*-value	0.992	<0.001	<0.001	<0.001	<0.001	<0.001

^a–c^ Different superscripts within each column are significantly different (*p* < 0.05). SOD: superoxide dismutase, CAT: catalase, GSH: reduced glutathione, MDA: malondialdehyde, PC: protein carbonyl, 8-OHdG: 8-hydroxy-2-deoxyguanosine.

**Table 3:** Effect of supplementation of OEO (1 g/kg diet) on inflammatory biomarkers in rabbits exposed to AFB1 (0.3 mg/kg diet) for eight weeks.

Items.	Inflammatory Biomarkers			
	TNFα (pg/mL)	NO (nmol/µL)	VEGF (pg/mL)	TGF-β1 (pg/mL)
Control	174.29 ± 0.69 ^b^	87.11 ± 0.62 ^a^	4.42 ± 0.02 ^c^	12.29 ± 0.13 ^c^
OEO	169.50 ± 0.89 ^b^	87.19 ± 0.53 ^a^	4.03 ± 0.09 ^c^	9.64 ± 0.22 ^d^
AFB1	237.87 ± 2.46 ^a^	87.51 ± 0.17 ^a^	15.85 ± 0.27 ^a^	27.82 ± 1.09 ^a^
AFB1 + OEO	173.19 ± 0.29 ^b^	87.54 ± 0.48 ^a^	5.58 ± 0.36 ^b^	19.84 ± 0.83 ^b^
*p*-value	<0.001	0.891	<0.001	<0.001

^a–d^ Different superscripts within each column are significantly different (*p* < 0.05). TNFα: tumor necrosis factor α, NO: nitric oxide, VEGF: vascular endothelial growth factor, TGF-β1: transforming growth factor beta 1.

**Table 4:** Retention time and peak area (%) of the different compounds found in *O. vulgare* analyzed by GC-MS.

No.	Bioactive Chemical Constituents	RT (min)	Peak Area, %
1	19-Octanol	9.61	0.27
2	ci10s-trans Sabinene hydrate	10.33	3.47
3	Cycl11ohexanol	10.33	3.47
4	Terpinen-4-ol	12.03	1.24
5	Estragole	12.36	2.61
6	Thymol methyl ether	12.37	2.3
7	Carvacrol methyl ether	12.41	5.6
8	2,4-Decadienal	14.32	3.70
9	2-Pentene	15.72	1.77
10	Methyleugenol	16.45	0.06
11	Epiglobulol	18.48	0.48
12	Junipene	18.48	0.48
13	Naphthalene	18.62	0.37
14	Nerolidyl acetate	18.76	0.12
15	l-(+)-Ascorbic acid	29.11	16.11
16	5-Amino-2-methyl-2-phenyl-2	29.66	6.20
17	cis-13-Octadecenoic acid	32.63	32.63
18	Oleic Acid	32.63	48.57
19	Oleic acid, eicosyl ester	32.85	1.15

**Table 5:** Ingredients and composition of commercial growing rabbit diet (% of the as-fed diet).

Items	Basal Diet
Ingredient	
Soybean meal	15
Wheat bran	25
Berseem hay	30
Barely grain	28
Limestone	1
NaCl	0.5
Premix *	0.5
Total	100
Calculated composition, %	
Digestible energy, MJ/kg	10.85
Crude protein	17.29
Calcium	0.87
Phosphorus	0.54

* Each one kg of premix (minerals and vitamins mixture) contains vit. A, 20,000 IU; vit. D3, 15,000 IU; vit. E, 8.33 g; vit. K, 0.33 g; vit. B1, 0.33 g; vit. B2, 1.0 g; vit. B6, 0.33 g; vit. B5, 8.33 g; vit. B12, 1.7 mg; pantothenic acid, 3.33 g; biotin, 33 mg; folic acid, 0.83 g; choline chloride, 200 g.

**Table 6:** Primers sequences (forward and reverse) used for Real-Time qPCR studies.

Accession No.		Genes Sequence
NM_001101683.1	F: 5′-CTGGAACGGTGAAGGTGACA-3′ R: 5′-CGGCCACATTGCAGAACTTT-3′	β-actin
XM_002719559.3	F: 5′-CGTGGAGTCCTACACCTACAAC-3′ R: 5′-ACTCGTCTTTCTCGGCCATC-3′	HSP70
NM_001082263	F: 5′-CTGCACTTCAGGGTGATCG-3′ R: 5′-CTACGTGGGCTAGAGGCTTG-3′	TNF-α
NM_001082201	F: 5′-TTGAAGAAGAACCCGTCCTCTG-3′ R: 5′-CTCATACGTGCCAGACAACACC-3′	IL-1β
NM_001081991	F: 5′-TGCCAGGACACACTAACCAGAG-3′ R: 5′-TGTCACTCTCCTCTTTCCAATTCC-3′	IFN-γ

## Data Availability

The data presented in this study are available on request from the corresponding authors.
